# The Cervical Spine of the American Barn Owl (*Tyto furcata pratincola*): I. Anatomy of the Vertebrae and Regionalization in Their S-Shaped Arrangement

**DOI:** 10.1371/journal.pone.0091653

**Published:** 2014-03-20

**Authors:** Markus Krings, John A. Nyakatura, Martin S. Fischer, Hermann Wagner

**Affiliations:** 1 Institute of Zoology, RWTH Aachen University, Aachen, Germany; 2 Institut für Spezielle Zoologie und Evolutionsbiologie mit Phyletischem Museum, Friedrich-Schiller-Universität Jena, Jena, Germany; University of Lethbridge, Canada

## Abstract

**Background:**

Owls possess an extraordinary neck and head mobility. To understand this mobility it is necessary to have an anatomical description of cervical vertebrae with an emphasis on those criteria that are relevant for head positioning. No functional description specific to owls is available.

**Methodology/Principal findings:**

X-ray films and micro-CT scans were recorded from American barn owls (*Tyto furcata pratincola*) and used to obtain three-dimensional head movements and three-dimensional models of the 14 cervical vertebrae (C1−C14). The diameter of the vertebral canal, the zygapophyseal protrusion, the distance between joint centers, and the pitching angle were quantified. Whereas the first two variables are purely osteological characteristics of single vertebrae, the latter two take into account interactions between vertebrae. These variables change in characteristic ways from cranial to caudal. The vertebral canal is wide in the cranial and caudal neck regions, but narrow in the middle, where both the zygapophyseal protrusion and the distance between joint centers are large. Pitching angles are more negative in the cranial and caudal neck regions than in the middle region. Cluster analysis suggested a complex regionalization. Whereas the borders (C1 and C13/C14) formed stable clusters, the other cervical vertebrae were sorted into 4 or 5 additional clusters. The borders of the clusters were influenced by the variables analyzed.

**Conclusions/Significance:**

A statistical analysis was used to evaluate the regionalization of the cervical spine in the barn owl. While earlier measurements have shown that there appear to be three regions of flexibility of the neck, our indicators suggest 3–7 regions. These many regions allow a high degree of flexibility, potentially facilitating the large head turns that barn owls are able to make. The cervical vertebral series of other species should also be investigated using statistical criteria to further characterize morphology and the potential movements associated with it.

## Introduction

Birds have an S-shaped neck [1]–[3] with often a high degree of flexibility [4]–[7]. The cranial end of the neck is formed by the atlas that forms a joint with the occipital condyle. The neck ends caudally at the thorax, where the vertebrae have true ribs that connect to the sternum. According to Boas [2], the cervical column of birds may generally be divided into three natural regions (our translation of the word sequence “allgemein in drei natürliche Abschnitte zerfällt” used by Boas (p. 6)). Others have come to similar conclusions, although the number of identified functional regions varies across individual species and studies [3], [8]–[11]. These functional regions may be characterized by vertebral morphology and mobility.

Neck and head mobility in owls is interesting, because these birds are well known for their extraordinary degree of head mobility [12]–[14], which is even more interesting owing to the relatively short neck of owls [3]. A coarse analysis of the cervical vertebrae of the European barn owl (*Tyto alba*) that did not investigate how the different vertebrae interact was presented in [15]. Hivernaud [15] counted 12 cervical vertebrae, whereas other authors reported 13 [3], [16]. To our best knowledge no quantitative study with barn owls is available to evaluate regionalization (e.g., like that described in Ref. 2).

**Figure 1:**
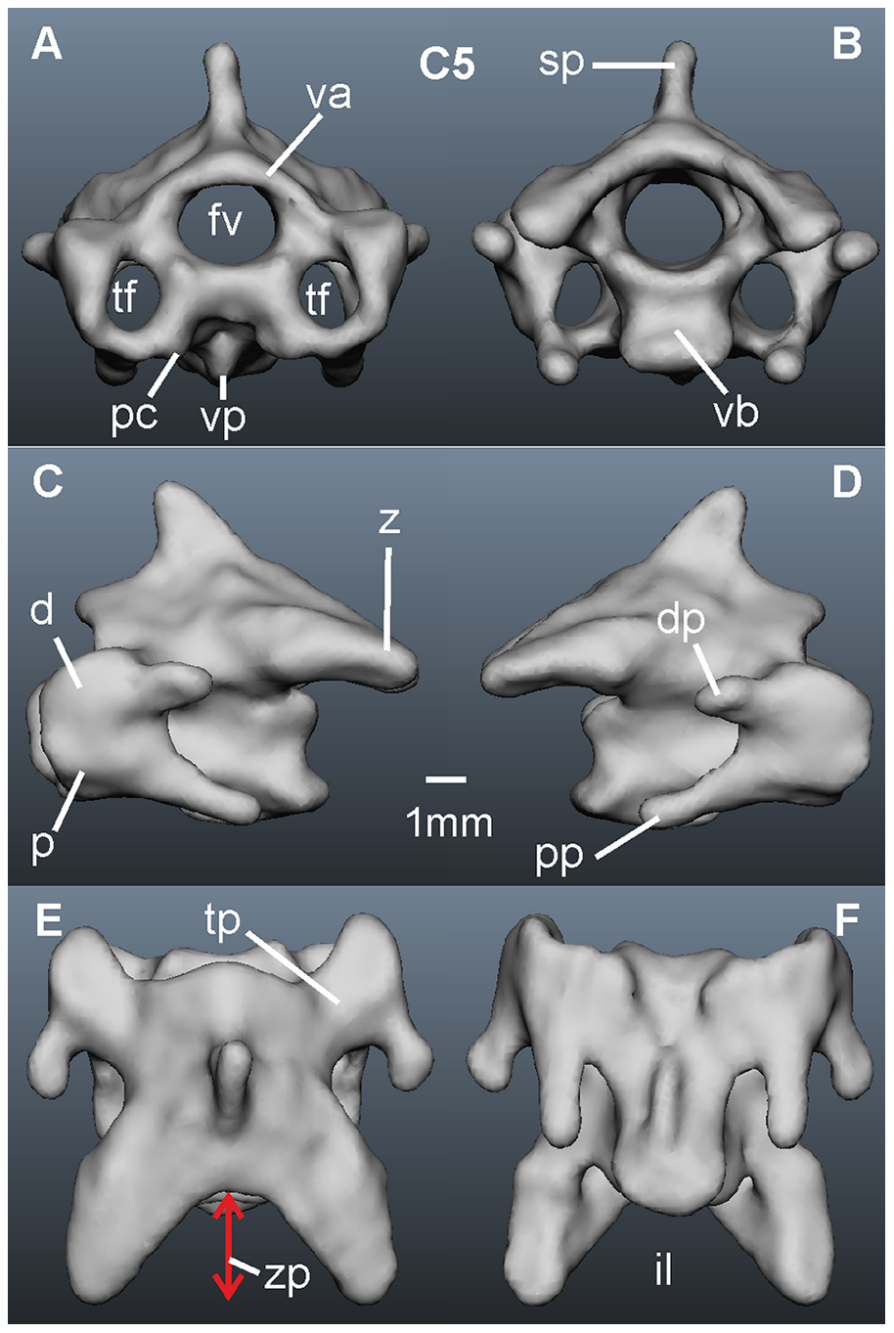
The anatomy of a vertebra. The fifth cervical vertebra of owl 1 (*Tyto furcata pratincola*; adult female, weight: 500 g) is used for demonstration here. A) Cranial view. B) Caudal view. C) Left lateral view. D) Right lateral view. E) Dorsal view. F) Ventral view. Marked are the ventral body (vb, *corpus vertebrae*), the vertebral arch (va, *arcus vertebrae*), the vertebral canal (fv, *foramen vertebrale*), the spinous process (sp, *processus spinosus*), the ventral process (vp, *processus ventralis corporis*), a transverse process (tp, *processus transversus*), a postzygapophysis (z, *zygapophysis caudalis*), the transverse or arterial canals (tf, *foramina transversaria*), a diapophysis (d), a parapophysis (p), a diapophyseal process (dp), a parapophyseal process (pp), a *processus caroticus* (pc), and the *lacuna interzygapophysealis* (il). The length of the *lacuna* will be called zygapophyseal protrusion (zp (see red double arrow in E)).

Not only the number of vertebrae, but also the size and morphology of individual vertebrae are important variables underlying the mobility of the neck and head [3], [7], [17], [18]. Knowledge of the morphology of the vertebrae may allow for delineating possible movements, but care is necessary, because the surrounding tissue also influences neck mobility [7]. A typical cervical vertebra is displayed in Figure 1, and described following definitions in [19]. Briefly, a core structure formed by a vertebral arch (*arcus vertebrae*) and a vertebral body (*corpus vertebrae*) surround the vertebral canal (*foramen vertebrae*), an opening through which the spinal cord runs. The thickness of the spinal cord sets a lower boundary for the diameter of the vertebral canal, and shearing movements during head rotation may impose further limitations on its size. For these reasons the diameter of the vertebral canal was used as the first criterion in our study. Two more canals (*foramina transversaria*) are often present and serve as openings for blood vessels and pneumatic diverticula. The transverse processes (*processus transversi*) arise on each side of the vertebral arch and project laterally. Further processes, called zygapophyses, originate at the base of the transverse processes and project anteriorly (prezygapophyses) or posteriorly (postzygapophyses) [3], [20]. The postzygapophyses articulate with the prezygapophyses of the next vertebra in the series to form synovial joints. The V-like notch between the postzygapophyses is the *lacuna interzygapophysialis*, the cranio-caudal extent of which, the zygapophyseal protrusion, is our second criterion for neck mobility (Figure 1E). The structure of the surfaces at the joints of most cervical vertebrae is saddle-shaped, which allows sliding at close distance while preventing disarticulation of vertebral articular faces during neck movement [6] and, therefore, the distance between the joint centers represents our third criterion. Lastly, cervical vertebrae exhibit some pitching in the natural posture that cumulatively imparts to the vertebral column a sigmoid shape. This pitching angle is the fourth criterion in this study.

We chose to study cervical vertebrae of the American barn owl (*Tyto furcata pratincola*) for several reasons: first, to assess variability in vertebral counts and find out why previous studies of barn owl differ in the number of cervical vertebrae reported [3], [16], second previous anatomical descriptions of the barn owl structures are coarse and restricted to the European barn owl [15], and, third, the individual cervical vertebrae of the barn owl have not been studied as a functional cervical column. We shall report that the cervical column may be divided into several regions according to cluster analysis done with the four variables mentioned above.

## Materials and Methods

This work is based on the data from 3 American barn owls (*Tyto furcata pratincola*, formerly *Tyto alba pratincola*) from the breeding colony of the Institute of Biology II at RWTH Aachen University, Aachen, Germany. Care and treatment of the owls complied with the NIH Guide for the use and care of laboratory animals. They were approved by the Landespräsidium für Natur, Umwelt und Verbraucherschutz Nordrhein Westfalen, Recklinghausen, Germany, and the Thüringer Landesamt für Lebensmittelsicherheit und Verbraucherschutz, Abteilung Gesundheitlicher Verbraucherschutz, Veterinärwesen, Pharmazie, Bad Langensalza, Germany.

### Data collection

**Figure 2:**
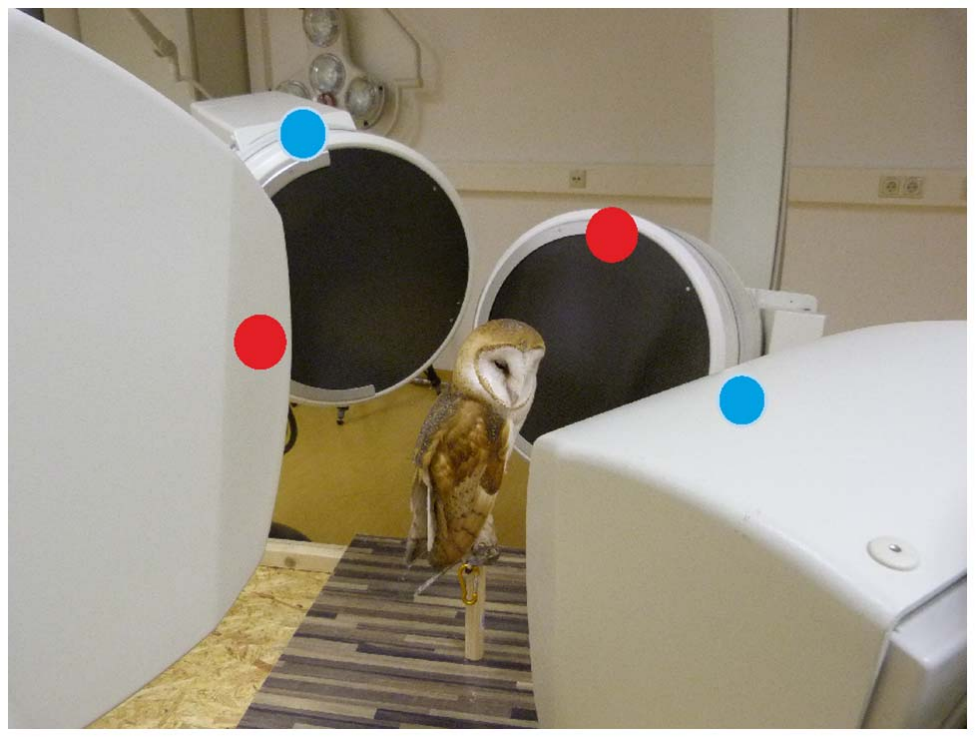
Photograph of an owl in the setup in Jena. The owl (*Tyto furcata pratincola*) is sitting on a perch with the head in the crossing point of the two X-ray systems (red and blue dots).

XMA technology (X-ray motion analysis) [21], [22] was used to visualize the head and neck movements of barn owls in three dimensions (Figure 2). Data were collected in the biplanar setup at Friedrich-Schiller-Universität Jena (Neurostar, Siemens, Munich, Germany; 50 kV) [23], allowing synchronized high-speed recordings (500 frames/s) from two perpendicular projections (SpeedCam Visario g 2; Weinberger, Dietikon, Switzerland). The pixel resolution was 1536×1024, resulting in an absolute spatial resolution of about 0.4 mm [24]. A third synchronized standard light camera (SpeedCam Visario, Weinberger, Dietikon, Switzerland) provided a more low-resolution view (512×512 pixel) of the scenery and the barn owl's movement. During the recordings an owl was placed on a T-shaped perch with the legs loosely tethered to the perch, but able to move freely otherwise (Figure 2). Spontaneous as well as induced head rotations were filmed. These sequences served as reference for the rearticulation of the cervical column (see below).

We dissected the cervical spine of two carcasses and isolated all vertebrae individually, as well as the occiput and the first thoracic vertebra. Digital three-dimensional micro-CT scans were obtained from the 14 cervical vertebrae, the occiput and the first thoracic vertebra of 2 dead animals (v|tome|x s, GE phoenix|x-ray (Wunstorf, Germany); voxelsize: owl 1: 104.8 μm, owl 2: 135.37 μm; rotational resolution: 1 degree). Three-dimensional models of the cervical vertebrae were reconstructed from the CT-scans with the help of the software Amira® (Mercury system, Chelmsford) and the software MeshLab® (ISTI-CNR, Pisa).

### Anatomically defined coordinate system

**Figure 3:**
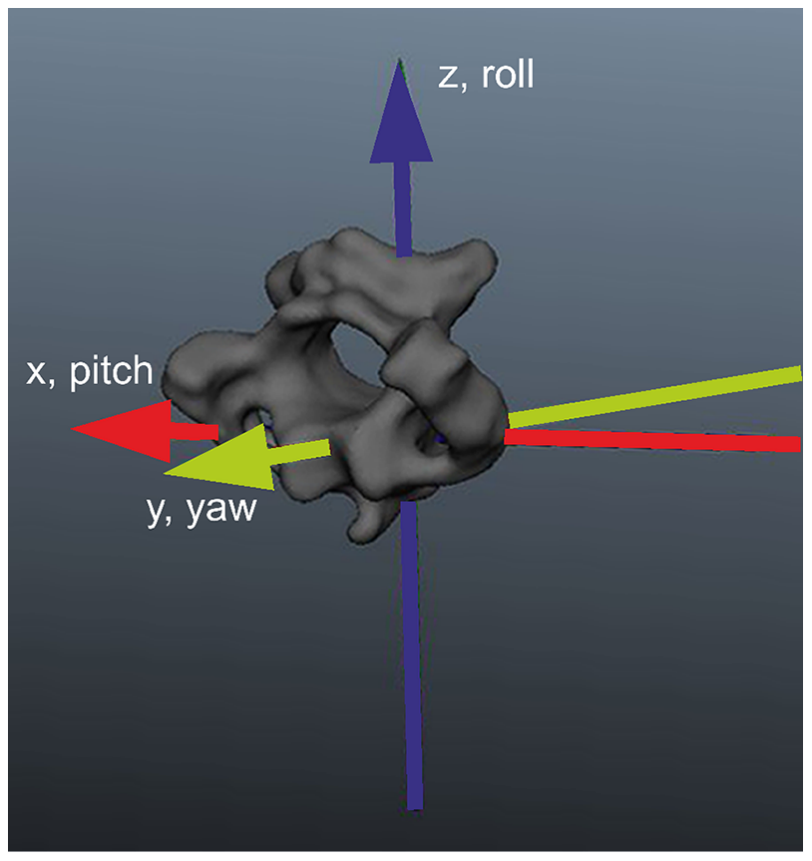
Coordinate system. The twelfth cervical vertebra is used for demonstration. The origin of the coordinate system is in center of an intervertebral joint. The x-axis is marked red, the y-axis green, and the z-axis blue.

The bone models were imported into the animation program Maya® (Autodesk, San Raphael). This software enabled us to reconstruct and rearticulate the vertebrae hierarchically by virtual intervertebral joints using a right-hand Cartesian coordinate system. The origin of the coordinate system was located in the center of an intervertebral joint (Figure 3). The x-axis was defined as the left-right axis with positive values to the right side of the body (Figure 3, red axis). The y-axis was defined as the cranio-caudal axis with positive values in the direction of the head (Figure 3, green axis). The z-axis was defined as the dorso-ventral axis (Figure 3, blue axis). Rotation about the x-axis will be termed pitch movement, rotation about the y-axis will be called longitudinal axis rotation or yaw movement, and rotation about the z-axis will be referred to as lateral bending or roll movement.

### Measurements indicating cervical mobility

Four criteria were used to judge cervical mobility: diameter of the vertebral canal, zygapophyseal protrusion, distance between joint centers, and pitching angle.

Diameter of the vertebral canal (see below): The vertebral canal is elliptical. It was observed in viewing the X-ray films that lateral bending and longitudinal axis rotation, the two main rotational movements during head rotation, induce shearing between successive vertebrae and, thus, decrease the effective medio-lateral diameter of the vertebral canal much more than the vertical one. In a similar way the vertebral canal is also related to movability in humans [25]. Therefore, it was decided to use the maximum medio-lateral diameter of the vertebral canal as the first criterion.

Zygapophyseal protrusion (see Figure 1 and below): To determine the extent of the zygapophyseal protrusion, the two end points of the postzygapophyses were connected by a line. A second line was drawn perpendicular to the first line and positioned so that it ended in the center of the vertebral arch. The distance between the end of the vertebral arch and the intersection point of the two lines was the zygapophyseal protrusion, measured in mm. Note that this determination of the zygapophyseal protrusion differs from the zygapophyseal overlap used by others [18] in that it used the anatomy of one vertebra only and does not take into account the joint between two vertebrae.

Distance between joint centers (see also below): First, a coordinate system was positioned in the center of the main joint of each vertebra. Second, the two vertebrae in consideration were positioned so that the counterparts of the respective joints (both the main joint and the zygapophyseal joints) were facing each other. Last, the vertebral column was aligned so that both the orientation of the vertebrae as well as the distance between the vertebrae best approximated the shape as seen in the X-ray films. The distance between the origins of the coordinate systems was measured and taken as the distance between joint centers. Control measurements before and after alignment showed deviations in distances were below 10%.

Pitching angle: This is the rotational movement about the x-axis measured in degrees with ventral bending corresponding to negative angles. The angle was always measured from one vertebra to the next cranial one.

### Cluster analysis

The calculation of the clusters was done with the Matlab (The Mathworks, Natick, MA, USA) built-in function *kmeans(X, k)*, *w*ith *X* as the varying dataset and *k* as the number of clusters [26]. The number of clusters was varied from 2 to 5. Each cluster analysis was repeated 1000 times. The output of the analysis is a vector which contains the relative frequency or percentage with which a given vertebra is associated with a cluster of a given number.

## Results

This work is based on results from X-ray films and micro-CT scans. Micro-CT scans were obtained from 2 owls, designated owl 1 and owl 2 in the following text, in Bonn, Germany. X-ray films were obtained from 2 owls, owl 1 and owl 3, in Jena, Germany, and analyzed in Jena and Aachen. Note that owl 1 was used for both X-ray filming and micro-CT scans. In the following, we first present a brief overview over the cervical vertebral column of the American barn owl, followed by a description of individual cervical vertebrae. For the measurement of the distance between joint centers and the pitching angle we positioned the vertebrae in a manner consistent with the natural shape of the neck as observed in the X-ray films. Finally, we conducted cluster analysis to identify potential morphological and functional regions in the cervical vertebral column.

### The shape of the neck of the American barn owl

**Figure 4:**
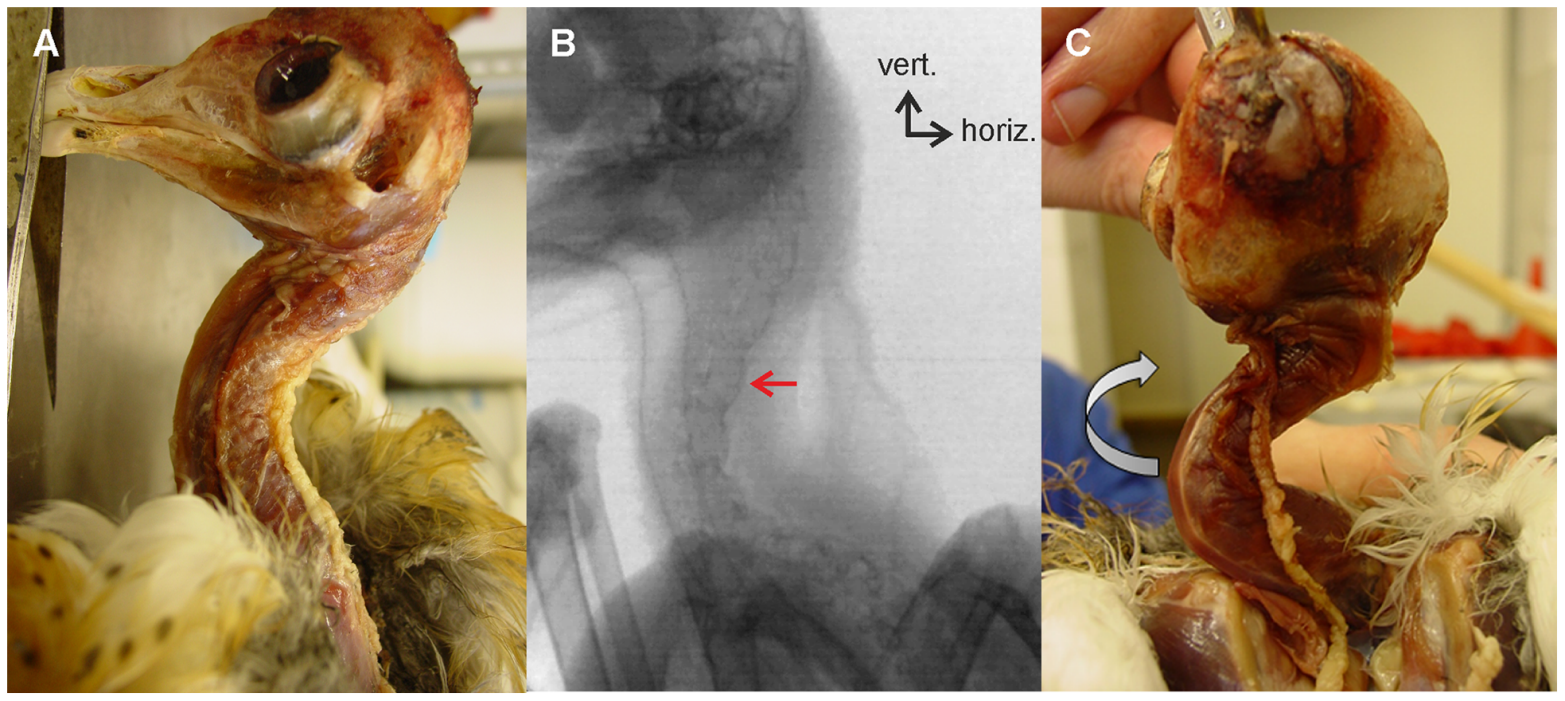
The neck of the barn owl. A) Cadaver of a barn owl in a left lateral view with the head on the left hand side. The plumage and skin of head and neck are removed. B) An X-ray picture of the neck of a living owl. One cervical vertebra is marked by a red arrow. The inset (vert = vertical; horiz = horizontal) indicates that the owl is sitting in an upright position. C) The same situation as in A) after rotating the head by 180°. Note that in the photograph in C) the corpus of the owl is rotated by 90 degrees about the vertical axis compared with the photograph in A). Note the S-shaped form and the contribution of all three regions to the rotation.

Figure 4 displays the neck of an American barn owl after the feathers were removed. Note the S-shaped form of the neck (Figure 4A). In Figure 4B, the neck and the vertebral column are visualized using a snapshot from an X-ray film in which the owl is seen in a typical upright position. Individual vertebrae are clearly visible in the X-ray films (red arrow in Figure 4B), revealing that the sigmoid shape of the vertebral column is a consequence of vertebral orientation. A forced rotation of the head by 180 degrees (Figure 4C) demonstrates that the rotational ability is not exclusively located in the cranial cervical spine, because torsion is also observed in the caudal part. Whereas individual vertebrae can be discriminated in the X-ray films, the resolution was not detailed enough to allow high-detailed reconstruction by the XROMM technology (scientific rotoscoping) [22].

### Reconstruction of the vertebrae from Micro-CT scans

**Figure 5:**
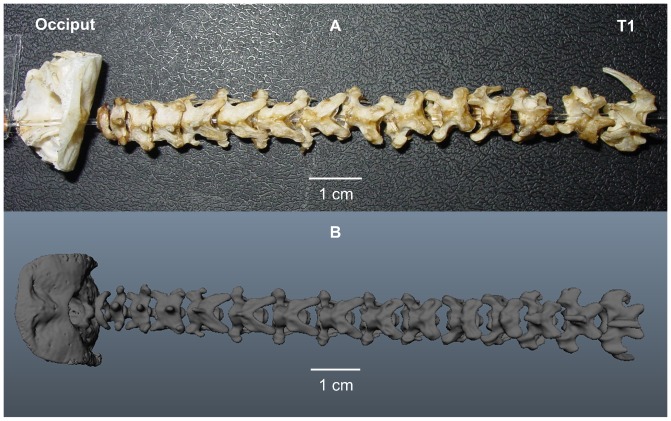
Reconstruction of the neck from micro-CT scans. A) Photograph of the cervical column in dorsal view, with the individual vertebrae threaded together from the occiput to the first thoracic vertebra (T1). B) Photograph of the reconstructed computer models of the cervical vertebrae in dorsal view arranged into a cervical column.

We dissected out all individual vertebrae, the occiput, and the first thoracic vertebra (Figure 5A), counting 14 cervical vertebrae. Discrimination between thoracic vertebrae and the cervical vertebrae was based on ribs, as ribs connecting to the sternum distinguish anterior thoracic vertebrae. Osteological data of the two owls (owls 1 and 2) are very similar (Table 1); as such, only data from owl 1, the owl used for both experiments, will be discussed in the following and used in the alignment. Whereas Figure 5A shows a photograph of the vertebrae assembled on a thread from the occiput to the thorax, Figure 5B shows a similar assembly of the 3-D computer models of the identical vertebra. Given the fragility of the actual specimens, we used strictly the computer models for reconstruction, as the digital models could be manipulated much easier than the delicate bones. In the coarse presentation shown in Figure 5, it may be seen that the size of the vertebrae changes from the small most cranial vertebrae, the atlas and the axis, to the middle and caudal ones. It is also obvious that the atlas has the shortest cranio-caudal extent or length (for a general description of a vertebra and the coordinate system used for the morphological descriptions see Figures 1 and 3). The cervical vertebrae in the middle have the largest cranio-caudal extent, especially because of their extended postzygapophyses, whereas the most caudal vertebrae are the widest ones (Table 1).

**Table 1:** Anatomical data of the cervical vertebrae.

Vertebra	Owl	Zygapophyseal protrusion (mm)	Diameter of vertebral canal (mm)	Distance between joint centers (mm)*	Height (mm)	Width (mm)	Length (mm)
C1	1	2.2±0.1	4.1±0.1	3.8	7.2±0.4	8.0±0.2	2.8±0.4
	2/3	1.6±0.1	3.8±0.1	3.7	6.9±0.4	7.0±0.2	2.2±0.4
C2	1	0.9±0.1	3.2±0.1	2.7	9.9±0.4	9.1±0.2	5.2±0.4
	2/3	0.9±0.1	3.0±0.1	2.6	9.7±0.4	10.0±0.2	5.0±0.4
C3	1	1.13±0.1	2.9±0.1	4.0	9.6±0.4	9.9±0.2	7.1±0.4
	2/3	1.2±0.1	2.7±0.1	4.0	9.0±0.4	9.6±0.2	7.5±0.4
C4	1	0.9±0.1	2.5±0.1	5.5	9.5±0.4	9.5±0.2	8.5±0.4
	2/3	1.2±0.1	2.6±0.1	4.4	8.8±0.4	8.6±0.2	7.8±0.4
C5	1	3.4±0.1	2.5±0.1	5.9	8.2±0.4	10.2±0.2	9.5±0.4
	2/3	4.1±0.1	2.4±0.1	5.7	7.9±0.4	10.2±0.2	9.4±0.4
C6	1	4.5±0.1	2.6±0.1	7.4	7.6±0.4	11.5±0.2	11.0±0.4
	2/3	4.4±0.1	2.6±0.1	7.4	6.8±0.4	10.9±0.2	10.5±0.4
C7	1	4.4±0.1	2.7±0.1	7.7	7.2±0.4	11.4±0.2	10.9±0.4
	2/3	4.1±0.1	2.5±0.1	7.9	7.3±0.4	10.8±0.2	10.0±0.4
C8	1	4.0±0.1	2.5±0.1	7.7	7.6±0.4	11.5±0.2	10.5±0.4
	2/3	4.4±0.1	2.4±0.1	7.8	7.4±0.4	10.6±0.2	10.1±0.4
C9	1	2.6±0.1	2.7±0.2	7.7	7.4±0.4	11.1±0.2	9.7±0.4
	2/3	4.0±0.1	2.5±0.1	7.0	8.0±0.4	10.2±0.2	9.3±0.4
C10	1	1.4±0.1	2.8±0.1	7.7	7.8±0.4	10.6±0.2	10.0±0.4
	2/3	2.1±0.1	2.7±0.1	7.6	7.9±0.4	9.9±0.2	8.0±0.4
C11	1	1.4±0.1	3.1±0.1	7.2	8.9±0.4	10.2±0.2	8.7±0.4
	2/3	1.5±0.1	2.9±0.1	7.2	8.8±0.4	9.8±0.2	7.8±0.4
C12	1	0.9±0.1	3.4±0.1	6.9	10.1±0.4	10.8±0.2	8.8±0.4
	2/3	1.6±0.1	3.6±0.1	6.6	9.3±0.4	10.4±0.2	8.1±0.4
C13	1	1.7±0.1	3.8±0.1	6.0	12.1±0.4	12.5±0.2	9.3±0.4
	2/3	1.7±0.1	4.0±0.1	6.0	11.5±0.4	12.7±0.2	8.9±0.4
C14	1	1.7±0.1	3.7±0.1	5.7	12.7±0.4	13.4±0.2	9.9±0.4
	2/3	1.7±0.1	3.8±0.1	5.7	13.2±0.4	13.4±0.2	8.7±0.4

* These data were taken from owl 3, whereas the other data were taken from owl 2.

**Figure 6:**
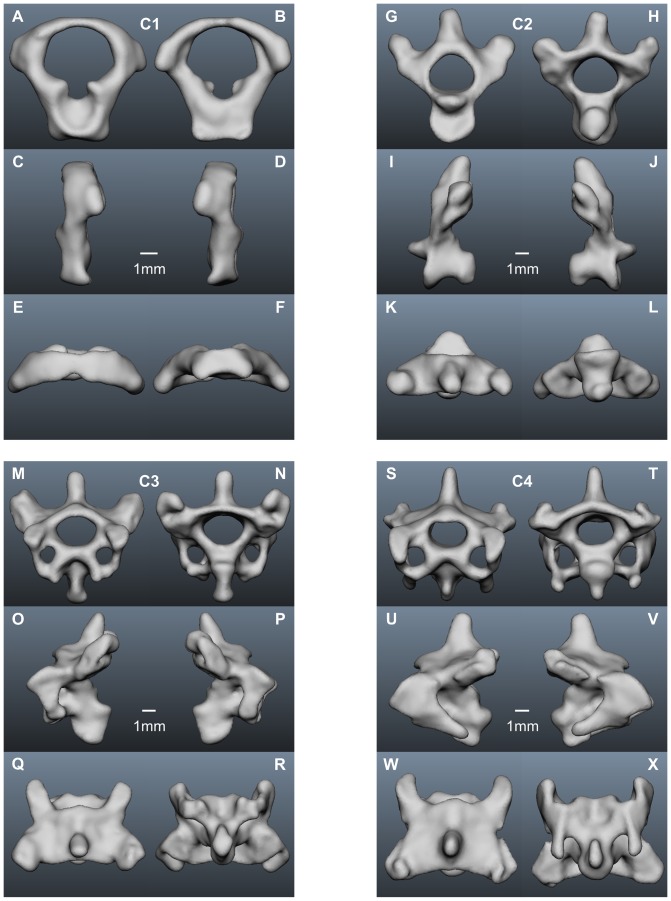
Cervical vertebrae of the American barn owl I. A–F) atlas; G–L) axis; M–R) 3^rd^ cervical vertebra; S–X) 4^th^ cervical vertebra. The length of the scale bar is constant for each vertebra, in other words the scale bar in D) refers to A–F. Please refer to the legend to Figure 1 for the orientation of the vertebra in the different images.

**Figure 7:**
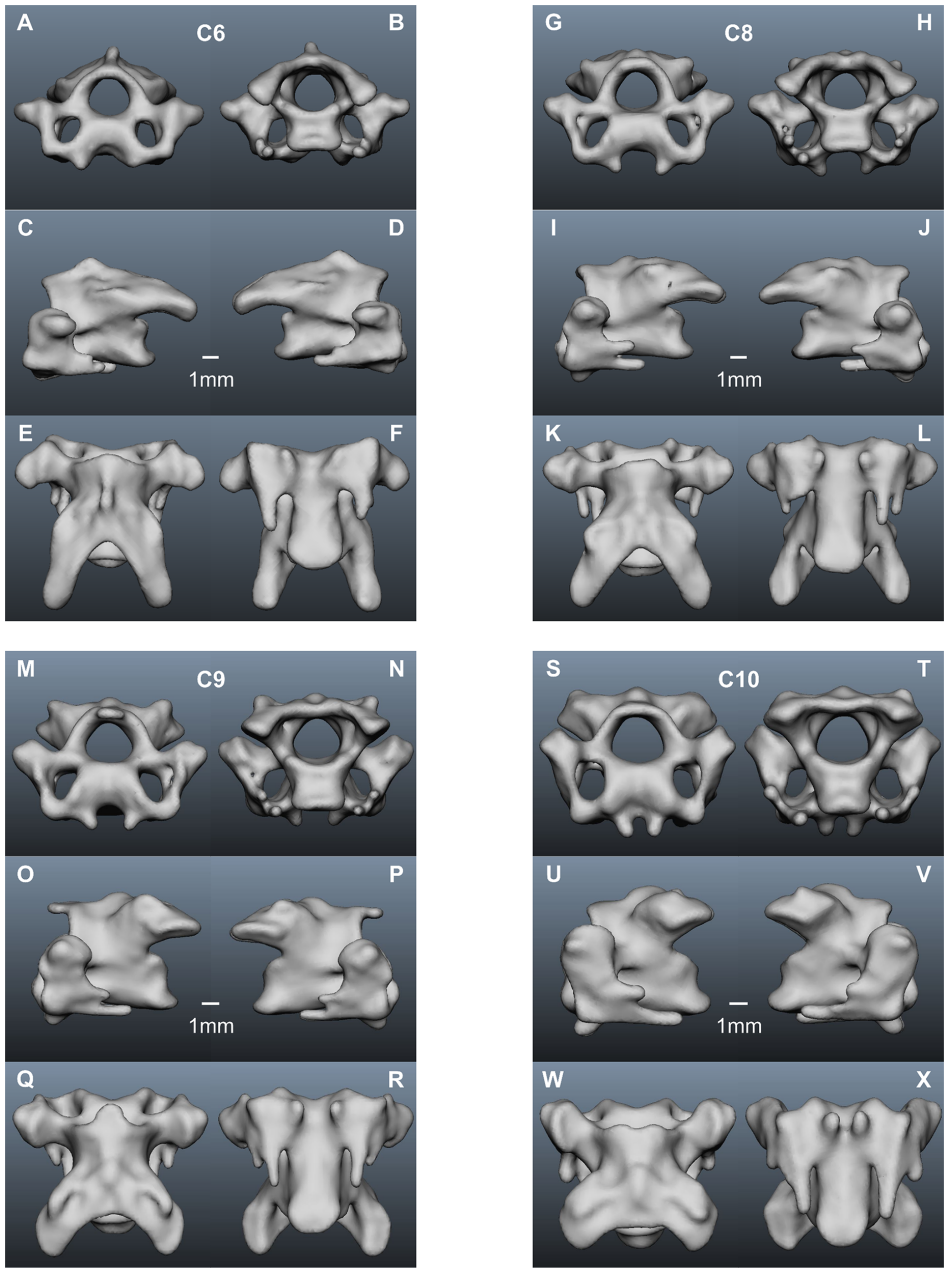
Cervical vertebrae of the American barn owl II. A–F) 6^th^ cervical vertebra; G–L) 8^th^ cervical vertebra; M–R) 9^th^ cervical vertebra; S–X) 10^th^ cervical vertebra. For further explanations see legends to Figures 1 and 6.

**Figure 8:**
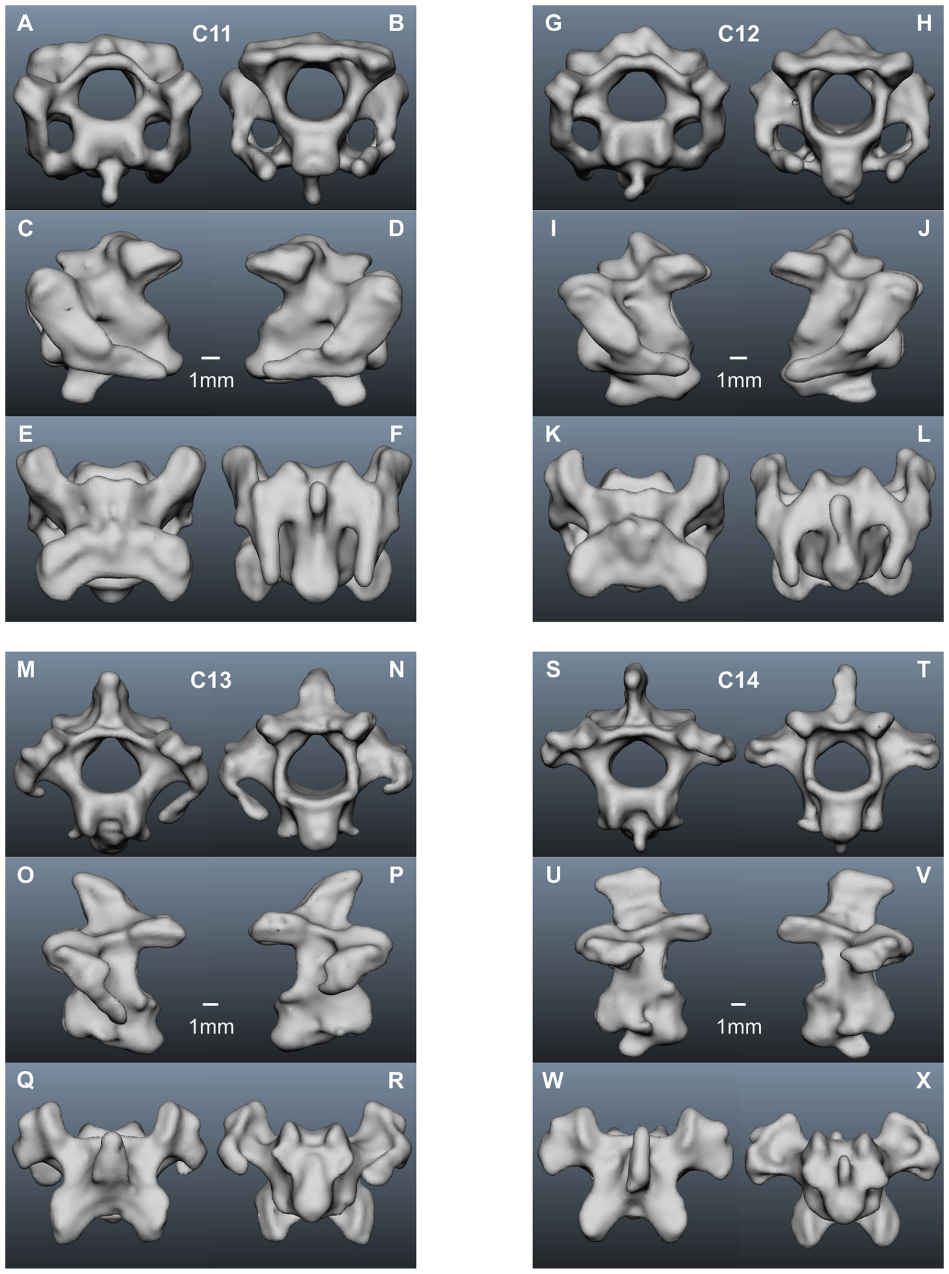
Cervical vertebrae of the American barn owl III. A–F) 11^th^ cervical vertebra; G–L) 12^th^ cervical vertebra; M–R) 13^th^ cervical vertebra; S–X) 14^th^ cervical vertebra. For further explanations see legends to Figures 1 and 6.

In the following we present a detailed description of the anatomy of all 14 vertebrae (see Table 1 for a summary). The computer model of each vertebra is shown in 6 views, cranial and caudal, left lateral and right lateral, and dorsal and ventral (Figures 1, 6–8). For the description of each vertebra we adopted a standard structure, mentioning first the size of a vertebra, than the size of the vertebral canal, the existence of transverse foramina, the existence and form of the different processes, and the size of the zygapophyseal protrusion.

The first cervical vertebra, C1 or atlas (Figure 6A–F), is the smallest of the cervical vertebrae (Table 1) (Figure 6A–F). The vertebral canal is very large (Figure 6A, Table 1). There are no transverse foramina for crossing arteries in C1 (Figure 6A, B). The simple shape of C1 shows no prominent processes: a spinous and a ventral process are missing, and the postzygapophyses are barely visible (Figure 6C–F). This also means that the zygapophyseal protrusion is very small (Table 1).

The second cervical vertebra, C2, the axis (Figure 6G–L), is larger than the atlas (Table 1). The diameter of the vertebral canal is smaller in size than that of C1 (Table 1). The axis has no arterial canals. A spinous process exists (Figure 6G, H). A ventral process exists as well (Figure 6G, H). The postzygapophyses are a little shorter than in C1 (compare Figure 6E–F with K–L). The zygapophyseal protrusion is very short (Table 1).

The third cervical vertebra, C3 (Figure 6M–R) is bigger in length than the first two vertebrae (Table 1). The diameter of the vertebral canal decreased compared with C2 and C1 (Table 1). C3 is the first cervical vertebra with arterial canals (Figure 6M, N). The spinous process is not directed vertically up as it is in C2, but is bent backwards (Figure 6P, Q). The ventral process is bigger in size than in C2 (Figure 6M, N). Transverse processes are clearly visible. The postzygapophyses are bigger in size than in C2 (Figure 6P, Q). Furthermore, left and right of the ventral process a *processus caroticus* is present (Figure 6M). The zygapophyseal protrusion has grown in size compared with C1 and C2 (Table 1).

The fourth cervical vertebra, C4 (Figure 6S–X), is nearly of the same size as the third one (Table 1). The diameter of the vertebral canal is the smallest of the first four cervical vertebrae (Table 1). C4 has arterial canals (Figure 6S, T). The spinous process differs from that of C3 by being directed more vertical up, comparable to the one of C2 (Figure 6S–V). The ventral process is still large (Figure 6S, T). Furthermore, C4 is the first cervical vertebra whose transverse processes are is clearly divided into a diapophysis and a parapophysis (Figure 6U, V). The parapophysis bears a big process which points caudally (Figure 6U, V). Very small *processus carotici* appear (Figure 6S). The zygapophyseal protrusion is still short (Table 1).

The fifth cervical vertebra, C5 (Figure 1A–F), is bigger in length and width than the first four cervical vertebrae (Table 1). The diameter of the vertebral canal is comparable to the one of C4 (Table 1). Here a difference between the two owls was observed: in owl 2 the vertebral canal of C5 was a little smaller than that of C4, whereas it was of the same size in owl 1 (Table 1). *Foramina transversaria* are present (Figure 1A, B). The spinous process is smaller in size than in C4 and is bent a little cranially (Figure 1C, D). As a consequence C5 is less high than C2, C3 and C4 (Table 1). The ventral process has become smaller than in C4, while the *processus carotici* are of medium size (Figure 1A). C5 is the first cervical vertebra with a diapophyseal and a parapophyseal process (Figure 1C–F). The parapophyseal process differs from the one of C4, because it shows first hints of having two peaks (Figure 1C, D). The postzygapophyses of C5 are much bigger than those of the first four cervical vertebrae (Figure 1A–F), and the zygapophyseal protrusion has grown in size (Table 1).

The sixth cervical vertebra, C6 (Figure 7A–F), is smaller in height but larger in width than C5 (Table 1). Moreover, C6 is the longest of all cervical vertebrae (Table 1). The diameter of the vertebral canal is at the same level as C4 and C5 (Table 1). The *foramina transversaria* are large (Figure 7A). The spinous process is very small (Figure 7A–F). C6 has no ventral process; instead a *sulcus caroticus* is present flanked by the *processus carotici* (Figure 7A, B). The diapophyseal process is smaller in size than the one of C5, whereas the second peak of the lower parapophyseal process is much more distinct (Figure 7A–D). The zygapophyseal protrusion has further grown in size (Table 1).

The dimensions and shape of the seventh vertebra, C7, are very similar to those of the neighboring cervical vertebrae (Table 1, Figure 7A–L). Therefore, no computer generated images of C7 are displayed. Table 1 also shows that the zygapophyseal protrusion is in the same range as the one in C6 as is the diameter of the vertebral canal (Table 1).

The eighth cervical vertebra, C8 (Figure 7G–L), is comparable to C6 in nearly all anatomical measures (Table 1). The vertebral canal of C8 is the narrowest of all cervical vertebrae (Table 1). The vertebra has transverse foramina (Figure 7G, H). The processes look similar to those of C6, and the zygapophyseal protrusion has a similar size than that of C6 (Table 1).

The ninth cervical vertebra, C9 (Figure 7M–R), is a little smaller in width and length than C8 (Table 1). Its height is comparable to that of C8 (Table 1). The diameter of the vertebral canal is still small (Table 1). Transverse foramina are present (Figure 7M, N). The zygapophyses are the most prominent processes. However, the postzygapophyses (Figure 7O–R) and the second parapophyseal peak (Figure 7O, P) are shorter than the ones of C8. The *processus carotici* are a little closer together than those of C8 ((compare Figure 7M, N with Figure 7G, H). The zygapophyseal protrusion has decreased in size compared with C8 (Table 1).

The tenth cervical vertebra, C10 (Figure 7S–X), is shorter in length than C9, while the height and width of C10 are comparable to those of C9 (Table 1). The diameter of the vertebral canal has not changed much compared with C9 (Table 1). C10 has transverse foramina (Figure 7S, T). As in C9 only the postzygapophyses are prominent, but even these are shorter than in C9 (compare Figure 7O–R with Figure 7U–X). The second parapophyseal peak has also shrunken (Figure 7U, V). The diapophyseal process is barely visible in C10 (Figure 7U, V). The *sulcus caroticus* has become very narrow (Figure 7S, T). The zygapophyseal protrusion is clearly shorter than in C9 (Table 1).

The eleventh vertebra, C11 (Figure 8A–F), is higher but also shorter than C10 (Table 1). The diameter of the vertebral canal is bigger than in all the vertebrae before except the atlas and the axis (Table 1). Transverse foramina are present (Figure 8A, B). A prominent ventral process appears, while the spinous process is inconspicuous (Figure 8A, B). The postzygapophyses are shorter than in C10 (compare Figure 8A–F with Figure 7S–X). The second parapophyseal peak disappears completely. The zygapophyseal protrusion is short (Table 1).

There are not many differences between C12 (Figure 8 G–L) and C11, but C12 is a little bigger in size than C11 as documented by its length, width and height (Table 1). The diameter of its vertebral canal is bigger than in C11, too (Table 1). Transverse foramina are present (Figure 8G, H). Moreover, a spinous process appears again (Figure 8G–J), while the ventral process continues to be large. The zygapophyseal protrusion is comparable to C11 (Table 1).

The thirteenth cervical vertebra, (C13 (Figure 8 M–R), is bigger in size than C12 (Table 1). C13 has one of the biggest vertebral canals – at the same level as the one of C1 (Table 1). It has no transverse foramina (Figure 8M, N), and the parapophysis has decreased in size (Figure 8M–P). A short cervical rib appears. This rib does not articulate with the sternum. The rib is not visible in the pictures, because it was lost in the course of the dissection. C13 has a huge spinous process pointing cranially (Figure 8M, N). The ventral process of C13 is less distinct than that in C11 and C12 (Figure 8M–P). Additionally, *processus carotici* are found (Figure 8M, N), whereas the postzygapophyses (Figure 8O, P) and the zygapophyseal protrusion (Table 1) become quite short (Table 1).

The most caudal cervical vertebra, C14 (Figure 8S–X), is the biggest cervical vertebra in height and width (Table 1). Its length is comparable to that of C13 (Table 1). The vertebral canal is the third biggest of all cervical vertebrae (Table 1). Transverse foramina do not exist (Figure 8S, T). A cervical rib that was bigger than that of C13 was also lost in the course of the dissection. The spinous process is the biggest of all cervical vertebrae. It does not end in a peak, but plain like the spinous processes of the thoracic vertebrae (Figure 8S–V). A ventral process as well as *processus carotici* are found (Figure 8S, T). The zygapophyseal protrusion is short, similar to the one of C13 (Table 1).

### Quantification of characteristic variables along the cervical column

Each of the vertebrae is characterized by a vertebral canal, further canals, by processes, and by its size. What are the most important variables to describe the vertebrae in relation to neck mobility? We chose the vertebral canal that is most narrow for C6−C8, the zygapophyseal protrusion that is small at both ends (C1, C2 and C13, C14), the distance between joint centers that is largest in the middle, and the pitching angle that changes in a characteristic way along the cervical spine. Of course, other variables could also be used for the characterization, like the length of a vertebra or the existence of a *foramen transversarium*. But the length of a vertebra is less closely related to mobility than the distance between joint centers, and, therefore, we chose the latter and not the former. A *foramen transversarium*, on the other hand, is present from C3 to C12 and its presence or absence is, therefore, not very informative with respect to neck mobility.

**Figure 9:**
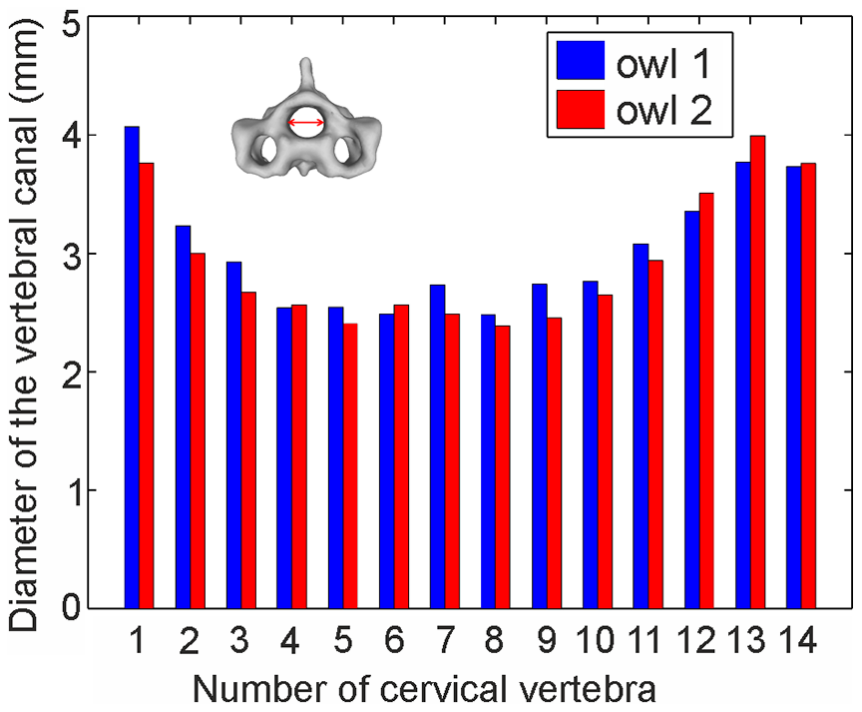
The diameter of vertebral canal. The inset shows how the diameter was measured. The measurements were conducted five times each, and the mean value is plotted. Note the decrease from the atlas to the central vertebrae and the subsequent increase towards the thorax.

We measured the diameter of the vertebral canal as a first characteristic morphological variable of the different vertebrae (Figure 9). The spinal cord has to run through the vertebral canal, and the diameter of the spinal cord sets a lower boundary for the diameter of the vertebral canal. In both owls, the diameter of the vertebral canal was much smaller in the middle region of the cervical spine than in the cranial and caudal segments (Figure 9). The largest values were around 4 mm, whereas the smallest values, in the middle of the cervical column, were around 2.5 mm. Since the spinal cord has to run through the canals of all vertebrae, it may be concluded that the spinal cord cannot fill the area provided by the cranial and caudal vertebrae. In other words, in the cranial and caudal vertebrae there is lots of room for movement of the spinal cord during rotation when shearing between the vertebrae may occur.

**Figure 10:**
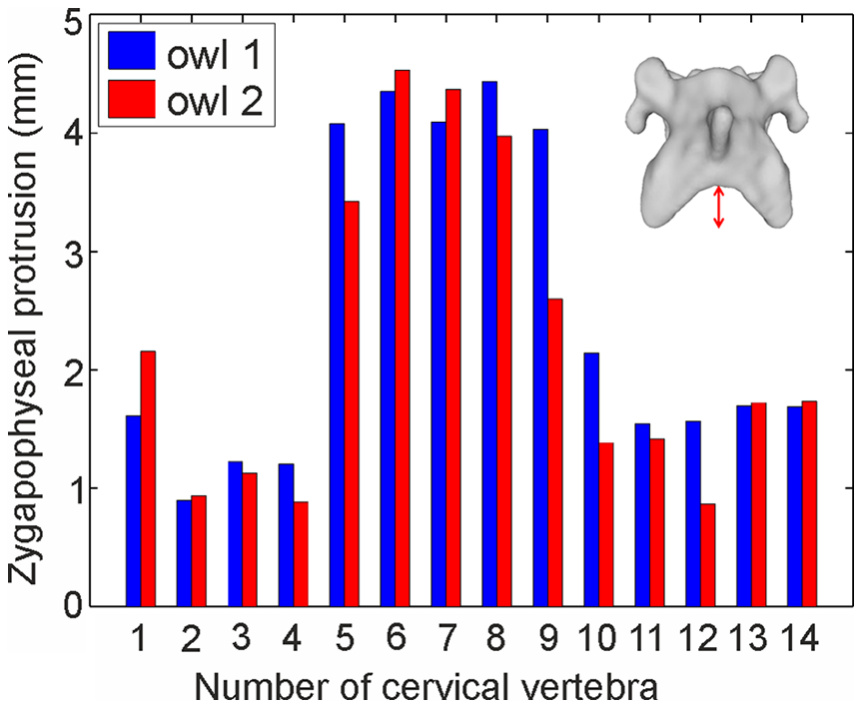
The zygapophyseal protrusion. The inset shows how the protrusion was measured. The measurements were conducted five times each, and the mean value is plotted. Note the big protrusion in the middle of the cervical column.

A second characteristic feature of the vertebrae is the zygapophyseal protrusion. The postzygapophyses overlap with neighboring vertebra, thus restricting joint mobility. The zygapophyseal protrusion was measured as the distance between a line connecting the end of the processes and the end of the vertebral arch (see inset in Figure 10, see also Materials and Methods). The zygapophyseal protrusion shows an almost inverse progression compared with the diameter of the vertebral canal (compare Figure 9 with Figure 10). The protrusion is much longer in the vertebrae from the middle region than in the vertebrae from the cranial and caudal regions of the neck. There is an abrupt increase between C4 and C5 in both owls. A difference between owl 1 and 2 was seen: in owl 1 the 9^th^ vertebra already shows a clear reduction in the zygapophyseal protrusion compared to the 8^th^ vertebra, with a smaller additional reduction between C9 and C10, whereas in owl 2, the biggest reduction occurred between C9 and C10.

The distance between joint centers and the pitching angle were chosen as the third and fourth variables characterizing neck mobility. These two variables require information about the joints and the interaction of vertebrae in a posture that is relevant for mobility. We turn to these issues in the next section.

### Description of the joints between the vertebrae

**Figure 11:**
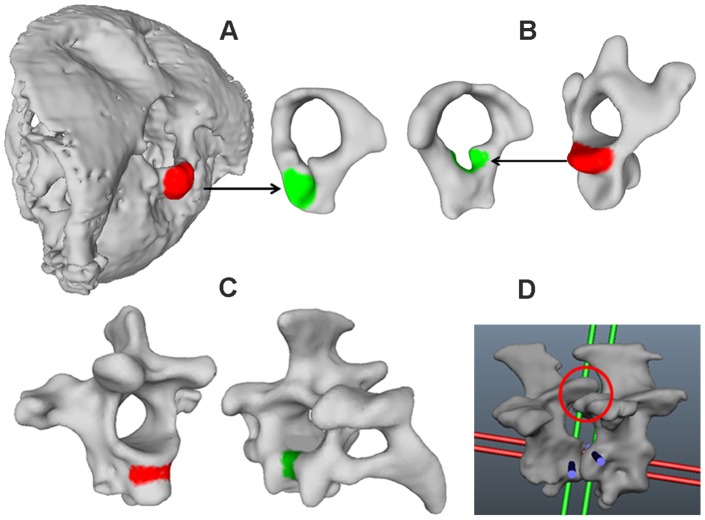
The joints and their alignments. A) The ball-and-socket joint between occiput (left) and atlas (right). The ball is marked green. The socket is marked red. B) The pivot joint between the atlas (left) and axis (right). The pivot is marked red. The ring-like socket is marked green. C) The saddle joint between C14 (left) and T1 (right). The convex area of C14 is marked red. The convex area of T1 is marked green. D) The saddle joint between C14 and T1. The two coordinate systems are shown. Additionally a zygapophyseal joint is marked with a red circle.

Types of joints were defined in accordance with MB Anatomy of Real Bodywork (Santa Barbara, California (USA)). The ball-shaped protrusion of the occiput (Figure 11A, red) articulates with a cavity-like socket of the atlas (Figure 11A, green) to form a ball and socket joint. The center of rotation in this joint is the center of the ball. To obtain the correct joint center, the ball was modeled as a sphere. The atlas and the axis form a pivot joint which consists of a pivot-like protrusion of the axis that fits into a ring-like socket in the atlas. The dens axis, with its lower curved surface and marked red in Figure 11B, is the pivot fitting in the socket (Figure 11, green) of the atlas. This curve was approximated by a sphere, and the joint center was positioned in the center of this sphere. Intervertebral joints of successive vertebrae in the series are semi-movable saddle joints (Figure 11C). Semi-movability means that the articulations are stabilized by a relatively inflexible layer of cartilage which is the intervertebral disc in this case. In a saddle joint both bones have a convex and a concave surface forming a saddle that interlocks with the saddle of the neighboring bone. The convex areas are colored in Figure 11C. Because of this specific structure the joint has two centers of rotation (Figure 11D): one in the center of each of the two convex areas. Both rotational centers were found by approximation of these areas with spheres. In summary, the atlas forms a ball-and-socket joint with the occiput and a pivot joint with the axis. This forms a saddle joint with C3 as do all the following vertebrae with each other.

**Figure 12:**
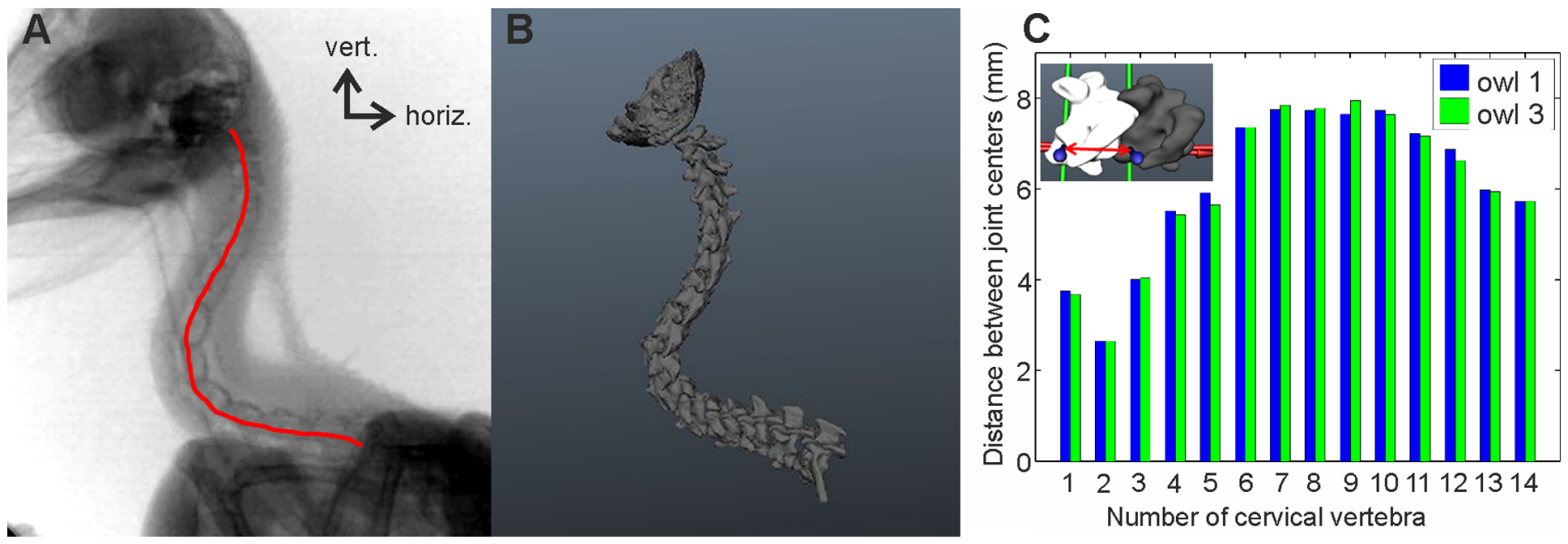
The cervical column in natural posture. A) X-ray image of owl 1 in the setup shown in Figure 2 displaying the natural posture. Note the S-shaped neck. The red line approximates the curvature of the cervical spine and was used for the realignment. B) Shape of the cervical column when the owl assumes its natural posture after realignment of the cervical vertebrae. The shape was extracted from the image shown in A). Note that the caudal part is almost horizontal, whereas the middle region is almost vertical. C) The distance between joint centers. The inset shows how the distance was measured. Joint 0 refers to the join between occiput and atlas, joint 1 between atlas and axis and so on. Note that the highest values occur in the middle of the cervical column.

The joints along the cervical column influence neck mobility, and the distance between joint centers is an important characteristic. We tested this by varying this distance and found that mobility was indeed correlated with distance (see supplementary figure). Therefore, we chose to examine the interaction of the vertebrae, and thus the distance between joint centers, from the X-ray films. This assumes that in these films, when the owl performs head rotations, the alignment of the cervical column reflects the natural, and, thus, veridical distance between joint centers. Twenty single frames of the X-ray films were chosen for owls 1 and 3, respectively. The owl was viewed by the camera from the side so that the two eye sockets were overlaying each other on the images (Figure 12A). The 20 images were compared and that image was chosen for further analysis that represented best the mean posture of all images. Next, the cervical column of the models of the vertebrae (Figure 5B) was aligned so that it best matched the shape of the cervical column in the chosen image (Figure 12B). Note the S-shaped form with an almost horizontal orientation of the caudal region of the vertebral column, a largely vertical orientation of the middle region and a slight ventral bending of the cranial region. The distance between joint centers and the pitching angle was measured from the alignment shown in Figure 12B in owl 1. For the measurements in owl 3, we proceeded as in the case of owl 1. However, we used the cervical model of owl 1 (Figure 5B), because owl 3 is still alive. This was justified, because there were only small differences between the vertebrae in the data of owls 1 and 2 as shown in Table 1.

The distance between joint centers was measured as the distance between the origins of the coordinate systems (Figure 12C, inset, red double arrow). Since the joints between occiput and atlas and between atlas and axis do not lie at the same height but on a diagonal line, the distance between the joint centers, albeit small, is much larger than the length of the atlas (Table 1). There was a gradual increase for the distance from both sides of the cervical column towards the center with vertebrae 5–10 being separated more from each other than the other vertebrae (Figure 12C).

Regarding our fourth criterion, the pitching angles of the middle cervical spine (joints 7–11) are more positive than the ones of the cranial and caudal cervical spine. Whereas the pitching angles are clearly negative for joints 0–5, the sixth and seventh joints seem to be intermediate as are joints 11 and 12. By contrast, joints 13 and 14 have clear negative values again.

### Cluster analysis

**Figure 13:**
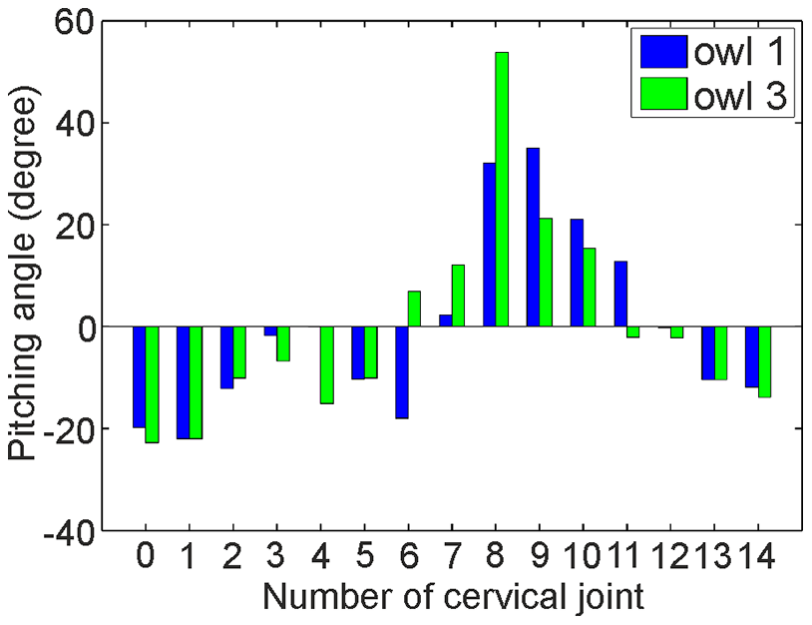
Pitching angles of owl 1 as measured from Figure 12B and of owl 3 as measured from a picture equivalent to Figure 12B (not shown).

We used the data from the diameter of the vertebral canal (Figure 9, Table 1), the zygapophyseal protrusion (Figure 10, Table 1), the distance between joint centers (Figure 12C, Table 1), and the pitching angle (Figure 13, Table 2) for a cluster analysis (for details see Materials and Methods). Whereas the first two criteria are purely osteological criteria, the latter are more movement related criteria taking into account interactions between vertebrae. Datasets for the zygapophyseal protrusion and the diameter of the vertebral canal stem from owls 1 and 2, whereas datasets for the distance between joint centers and the pitching angles stem from owls 1 and 3. As this means mixing datasets from different owls in the analysis, we were careful to test whether this mixing influenced the results using three different analyses. First, we only used the two osteological criteria, the diameter of the vertebral canal and the zygapophyseal protrusion, in the cluster analysis (analysis A). Second, we used only the movement related criteria, the distance between joint centers and the pitching angles, as criteria in the cluster analysis (analysis B). Third, we used all four criteria from owl 1 only (analysis C) and last from all three owls (analysis D). One variable in the cluster analysis is the number of clusters that is allowed. We allowed 2–5 clusters. Another variable is the initial setting. This is randomly chosen by the algorithm, and influences the formation of the clusters. Therefore, we ran the algorithm 1000 times and calculated the probability with which a vertebra was put into a given cluster.

**Table 2:** Pitching angles.

Joint		0	1		2	3	4	5	6	7
Pitching angle (deg)	Owl 1	−19.8	−22.0		−12.1	−1.8	−0.1	−10.3	−18.0	2.3
Pitching angle (deg)	Owl 3	−22.8	−22.0		−10.1	−6.8	−15.1	−10.1	6.9	12.1
Joint		8	9		10	11	12	13	14	
Pitching angle (deg)	Owl 1	32.1	35.0		21.0	12.8	−0.2	−10.4	−11.9	
Pitching angle (deg)	Owl 3	53.8		21.2	15.4	−2.1	−2.2	−10.4	−13.9	

**Figure 14:**
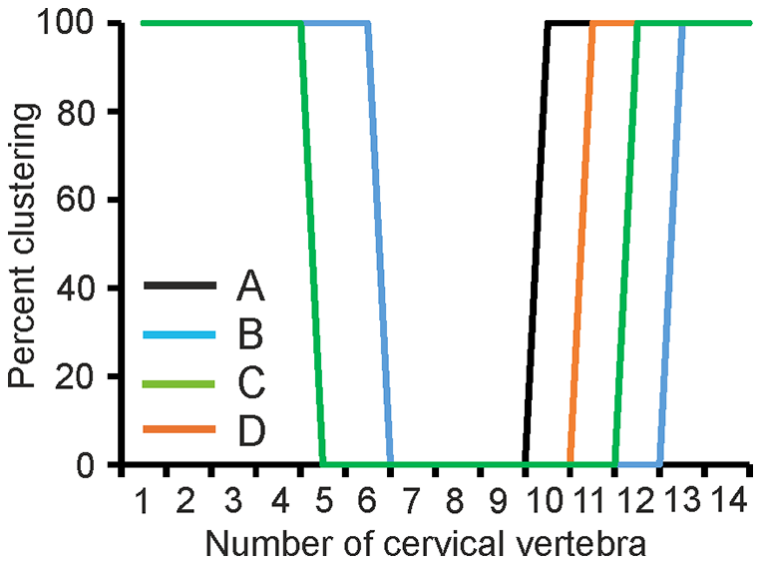
Results of cluster analyses with two clusters. Plotted are the percent of putting a given vertebra (x-axis) into a given cluster (y-axis) in the 4 different analyses (A–D, see inset). The clustering was always 100% or 0%. Only the results of cluster 1 are plotted. The results of cluster 2 are complimentary with 0% and 100% switched. Note that three regions could be detected with the 2-cluster analysis. Note also that the border between the clusters depended on the variables analyzed.

When two clusters were allowed the algorithm divided the cervical spine into three regions, a cranial, a middle and a caudal region (Figure 14). However, different borders between these three regions were suggested by the different analyses (A–D). This held specifically for the border between the middle and the caudal regions. Here each analysis found a different border. In analysis A, using the osteological criteria, the border lay between C9/C10, while in analysis B, using the movement related criteria, the algorithm located the border between C12/C13. The borders in the other two analyses lay in between these two extremes. By contrast, the border between the cranial and the middle region was more stable, with analyses A, C, and D yielding the same border – between C4 and C5. Only analysis B yielded a different border: here the clustering of C6 was split, with C6 in owl 1 clustering with the cranial vertebrae, whereas C6 in owl 3 clustered with the middle vertebrae. To us this result provided evidence for the existence of a regionalization, but also indicated that the borders may depend on the variables used.

**Figure 15:**
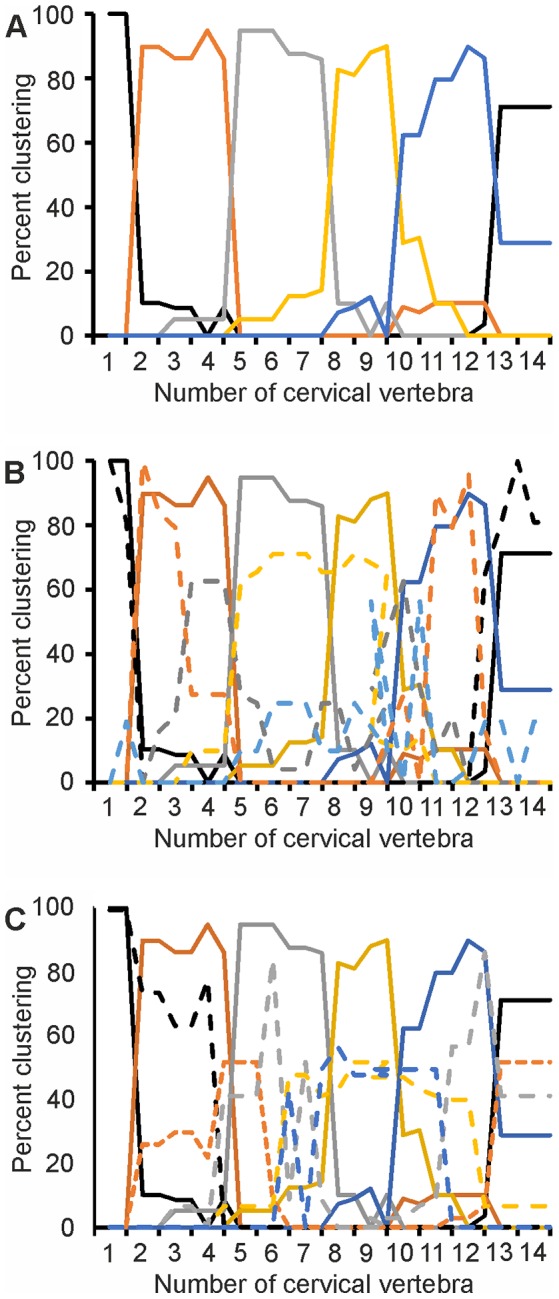
Results of cluster analyses with five clusters. A) All 4 variables were taken into account (analysis D). Note that 6 regions were found with the 5 clusters. B) Comparison of the clustering in analysis A (dashed lines) with that in analysis D (same data as in A)). Note that cluster borders shift between analyses. C) The same holds for a comparison of the clustering in analysis B (dashed lines) with that in analysis D. Note that in the 5-cluster-analyses, one vertebra was not always put into the same cluster in the different runs.

The results of the cluster analyses with more clusters were more complex. The clustering is shown in plots that document how often a certain vertebra was included in a given cluster and is expressed in percent (y-axes in Figure 15A–C). A high percentage shows a high confidence, whereas with a lower percentage confidence in the clustering drops. We show here data from the analyses with 5 clusters (Figure 15). The data with 3 and 4 clusters were in between the data with 2 and with 5 clusters. Analysis D with 5 clusters resulted in 6 regions (Figure 15A). Compared with the analysis with 2 clusters, the cranial region was split into two, with C1 standing apart from C2−C4. The middle region was also split in the 5-cluster analysis compared with the 2-cluster analysis. In the former analysis, the grey, the yellow and part of the blue clusters corresponded to the middle cluster of the latter analysis (compare Figure 15A with the orange line in Figure 14). The caudal region was now confined to C13 and C14. The results of analysis C (data not shown) were very similar to the results of analysis D.

Comparing the results of analysis D with those of analyses A and B for the 5-cluster cases, analysis A yielded a more divided regionalization than analyses B and D (Figure 15B, C). Whereas the cranial (C1) and caudal (C13+C14) regions were constant across analyses A, B and D (Figure 15, black lines), the more complex pattern in the 5-cluster analyses arose specifically in the transitional regions as suggested by the 2-cluster analyses. For example, the third region in analysis A in the 5-cluster case was located at C4 (grey dashed line in Figure 15B). Likewise, the fifth region in analysis A (light blue dashed line in Figure 15B) was located at C9 and C10 or between regions 4 (orange line in Figure 15B) and 5 (blue line in Figure 15B) as determined by analysis D. A similar picture arose when analyses B and D were compared. However, the transitional regions differed between the analyses: the most cranial transitional region in analysis B was at C5 (dark and orange dashed lines in Figure 15C) or between regions 2 and 3 as located in analysis D (orange and grey lines in Figure 15C). A second region of transition in analysis B was at C7 and C8 (see grey and blue dashed lines in Figure 15C). This region was not reflected in analysis D. Analysis B also suggested a transitional region between C11 and C12 (see blue, red and grey dashed lines in Figure 15C).

In our view, the data may be summarized in the following way: there is a regionalization in the cervical column, and the 2-cluster analysis found the tripartition already seen early on by others [2]. When more clusters are considered, the regionalization becomes more complex, not at the borders (C1 and C13/C14, respectively), but in the middle. The borders and the regionalization are influenced by the variables analyzed.

## Discussion

The anatomical description and the subsequent cluster analysis of the cervical vertebrae of the American barn owl yielded several regions with variable borders within the cervical spine. These findings will be discussed with respect to the anatomical shape of the vertebrae, the regionalization of the cervical spine, and the criteria to judge neck flexibility.

### The anatomical shape of the vertebrae

In general, the findings reported here regarding the anatomy of the barn owl's cervical vertebrae are in agreement with the existing literature [15]. However, there is no agreement in the literature with respect to the number of cervical vertebrae in the barn owl. We counted 14 cervical vertebrae in all the barn owls we studied, according to the definition that a cervical vertebra may have a false rib ending without attachment to the sternum, but not a true rib that attaches to the sternum [25]. This is in agreement with [14], but does not match with the number of 13 cervical vertebrae reported in [16]. It is unclear, why the latter authors did not find 14 cervical vertebrae in the same species. However, some variability in the number of cervical vertebrae is known from other vertebrates [27] and, thus, cannot be ruled out for *Tyto*. Twelve cervical vertebrae were described in [15] for *Tyto alba*, the European barn owl, a species closely related to the American barn owl. The reason for the difference between our counting and that of Hivernaud [15] might be that the latter author may have assumed that a cervical vertebra must not have any rib, be it a false or true rib. We found false ribs, also called cervical ribs, that do not attach to the breast bone in C13 and C14. So there are twelve cervical vertebrae without any rib in *Tyto furcata*. Cervical ribs are not only found in barn owls, but also in other birds including pigeons and chickens [28], [29]. In the general studies of the bird's skeleton by King and McLelland [28], it was claimed that the last cervical vertebra is fused with the first thoracic vertebrae. This was not the case in the specimens of *T. furcata* investigated here.

The cervical vertebrae of the barn owl displayed the general basic structure of a cervical vertebra consisting of a vertebral arch, a ventral body and a vertebral canal [19]. Apart from this, there were enormous differences in the shape of the processes between the cervical vertebrae. As was already mentioned in [6], transverse processes, arterial canals, ventral processes, or the *sulcus caroticus* varied between vertebrae. Articular facets at the processes could be found in all cervical vertebrae [6] with the exception of C1. Saddle shaped joints characterized the connections between the vertebrae caudally to the first two joints as also mentioned in [6]. The hollow, strut-stabilized structure of the cervical vertebrae was also reported in [29].

The data presented here compare well with the radiological and osteological atlas of the European barn owl presented in [15]. As judged from the photographs presented in this work, a big vertebral canal in C1 which lacks a dorsal and a ventral process is present in this species. Furthermore, the cavity-like socket for the articulation with the occiput and the ring-like socket for the dens axis can be seen. The features found in *T. furcata* match with the features on the photographs of *T. alba* for all cervical vertebra posterior to C2, save for C9 and C10, which are smaller in size than the preceding cervical vertebrae in *T. alba*. The *processus carotici* in *T. alba* are closer together than those in *T. furcata*. Likewise, no differences between T1 and T2 of *T. alba* as defined in [15] and C13 and C14 of *T. furcata* as defined here were observed. This supports our view that the cervical vertebrae with false ribs were counted as thoracic vertebrae in [15].

A specialized blood system of the owl's neck was reported in [14]. Our specimen also had the vertebral artery entering the cervical spine at C12 as indicated by the missing transversal canals in C13 and C14. De Kok-Mercado et al. [14] also report that the transversal canals are very spacious, providing more range of motion for the arteries, which agrees with our observations.

### The regionalization of the cervical spine

The results of the present study provide the most detailed description of the regionalization within the barn owl's cervical spine to date. Moreover, the data presented here may also have relevance for interpreting the regionalization of the bird neck in general. Since there is no objective criterion for determining a meaningful number of clusters, we varied cluster number in the statistical analysis. Interestingly, the data of the 2-cluster analysis are in agreement with the observations in [2] and recently in [3], [7], [10] of a tripartition of the cervical spine. However, if more clusters were allowed, more details were revealed. Such a more detailed regionalization was also proposed in [11]. In the barn owl, both the cranial and caudal regions are reduced to short segments, but these were constant across analyses. By contrast the middle regions were less stable and depended on the variable considered. We used criteria that were related to neck movement and measured the movement-related variables in the natural posture in our analyses similar to what was presented in [30] for mammals. This may have influenced the results, but we claim that it is important to use measures as closely to the natural situation as possible, if we want to understand the relation between morphology and function. It also seemed that the use of more variables like in analysis D resulted in a clearer picture than the use of fewer variables as in analyses A and B.

A partition for the genus *Tyto* was also presented in [3]. These authors considered the cervical spine of the barn owl to include 13 vertebrae and stated that the cranial region included C1 to C5, the middle one C6 to C9, and the caudal cervical spine C10 to C13. This classification differs from our results. One reason may be that a more subjective classification was used in [3] than here. The 2-cluster analysis applied here showed stable clusters of vertebrae at the cranial end (C1–C4), but variable middle and caudal regions. The 5-cluster analyses yielded an even more complex picture. Recent data of the cervical spine of penguins [11] indicated also more complexity in osteology. Thus, from an osteological point of view, more than 3 regions may exist. Our analysis A suggested 7 regions. The same holds for a movement-related perspective, although here the number of regions was only 6. A considerable overlap exists between the 6 modules reported in [11] and the regions we discriminate. Module 1 corresponds to our first region, comprised only of the atlas. Module 6 in penguins is similar to the most caudal region in the barn owl directly above the thorax. Differences exist in the middle of the cervical spine that is more homogeneous in penguins than in the barn owl. So the specializations of the penguins may lie in the center of the neck, which is sensible, because these birds “tuck in their neck while swimming” [11].

A description of the anatomy of the 13 cervical vertebrae found in the terror bird *Andalgalornis* was provided in [3]. These authors call the cranial cervical vertebrae short and robust. Although many metric properties are similar in *Andalgalornis* to those in the barn owl, we would not call the cranial vertebrae in the barn owl robust. The middle region in *Andalgalornis* displays big vertebral bodies and smaller spinous processes, comparable to the situation in the barn owl. By contrast, the middle vertebrae in *Andalgalornis* have a *sulcus caroticus* and not a ventral process as in the barn owl. Tambussi et al. [3] find that the sixth to eighth cervical vertebra in *Andalgalornis* are very similar which was also observed in the barn owl. The caudal region in *Andalgalornis* shows ventral processes, bigger spinous processes and cervical ribs in C12 and C13 [3]. Such a shape can be found in caudal cervical vertebrae of the barn owl as well.

### Relation of the anatomy of the vertebrae to neck flexibility

Whereas neck flexibility will be the theme of a separate publication (Krings et al., in preparation), we like to mention some general limitations imposed by the anatomy of the cervical vertebrae on neck flexibility here. Many authors assume a neutral pose of the neck, in which stress on the muscles, ligaments and joints is minimal [18], [30], [31]. We rearticulated the vertebrae to a cervical column using X-ray images for guidance. The rearticulation showed a dorsal pitching in the cranial region, a slight ventral pitching in the middle region, and a dorsal pitching in the caudal part. This is consistent with what has been reported for other species before [1]–[4], [31].

With respect to the relation between the anatomy of the vertebrae and neck flexibility, many criteria have been used for the determination of osteological limits of neck flexibility before, amongst them zygapophyseal overlap, centrum articular surface morphology, cervical rib length and neural spine orientation [7], [32]. Of this list of criteria we found the zygapophyseal overlap to be an important criterion with respect to our goal to understand head movements. We measured the effect of the zygapophyses on neck mobility by determining the size of the zygapophyseal protrusion. We think that the other criteria are not so closely related to neck mobility. By contrast, we decided to take the diameter of the vertebral canal as a criterion. The spinal cord, a very sensitive structure, runs through the vertebral canal. Since neck rotation is accompanied by shearing of the vertebrae, and since the integrity of the spinal cord needs to be maintained, the diameter of the vertebral canal should provide meaningful information about rotational limitations. Indeed, the four criteria we used were closely related to rotational limitations in the different regions of the barn owl's neck [33].

Cobley et al. [7] pointed out that estimations of neck functions based solely on osteological data should be viewed with caution. We agree with this statement. This was the reason why we carefully selected our criteria that included both the anatomy of the single vertebrae (diameter of the vertebral canal, zygapophyseal protrusion) and movement-related measures (distance between joint centers, pitching angle). We think that a combination of these criteria together with objective statistical analysis is best suited to reconstruct the veridical situation.

## Conclusions

Cluster analysis suggested that the cervical spine of the owl may be regionalized. Our indicators also suggested that the division into a cranial, a middle, and a caudal region may be too simple. Only the atlas and the two most caudal vertebrae were clustered in a very stably way in the different analyses. More variability existed along the rest of the cervical column as revealed by the four variables used here. The zygapophyseal protrusion, the diameter of the vertebral canal, the distance between joint centers, and the pitching angle form a complex set of data that suggests 6 or 7 regions along the cervical column.

## Supporting Information

Figure S1 **The maximum roll angle as a function of the relative distance of joint centers.** The distance of joint centers between C12 and C13 was varied, and the maximum roll angles were determined with the software Maya for every distance. Zero relative distance corresponds to the distance measured in the natural posture. Note the linear increase of the maximum angle with the distance of joint centers.
(TIF)
